# T-cell Receptor (TCR)-Peptide Specificity Overrides Affinity-enhancing TCR-Major Histocompatibility Complex Interactions*

**DOI:** 10.1074/jbc.M113.522110

**Published:** 2013-11-06

**Authors:** David K. Cole, Kim M. Miles, Florian Madura, Christopher J. Holland, Andrea J. A. Schauenburg, Andrew J. Godkin, Anna M. Bulek, Anna Fuller, Hephzibah J. E. Akpovwa, Phillip G. Pymm, Nathaniel Liddy, Malkit Sami, Yi Li, Pierre J. Rizkallah, Bent K. Jakobsen, Andrew K. Sewell

**Affiliations:** From ‡ Cardiff University School of Medicine, Heath Park, Cardiff CF14 4XN,; the § Medical Research Council Human Immunology Unit, Weatherall Institute for Molecular Medicine, University of Oxford, Oxford 0X3 9DS, and; ¶ Immunocore Ltd., 57C Milton Park, Abingdon OX14 4RX, United Kingdom

**Keywords:** Immunology, Major Histocompatibility Complex (MHC), Protein-Protein Interactions, Receptor Structure-Function, T-cell Biology, T-cell Receptor

## Abstract

αβ T-cell receptors (TCRs) engage antigens using complementarity-determining region (CDR) loops that are either germ line-encoded (CDR1 and CDR2) or somatically rearranged (CDR3). TCR ligands compose a presentation platform (major histocompatibility complex (MHC)) and a variable antigenic component consisting of a short “foreign” peptide. The sequence of events when the TCR engages its peptide-MHC (pMHC) ligand remains unclear. Some studies suggest that the germ line elements of the TCR engage the MHC prior to peptide scanning, but this order of binding is difficult to reconcile with some TCR-pMHC structures. Here, we used TCRs that exhibited enhanced pMHC binding as a result of mutations in either CDR2 and/or CDR3 loops, that bound to the MHC or peptide, respectively, to dissect the roles of these loops in stabilizing TCR-pMHC interactions. Our data show that TCR-peptide interactions play a strongly dominant energetic role providing a binding mode that is both temporally and energetically complementary with a system requiring positive selection by self-pMHC in the thymus and rapid recognition of non-self-pMHC in the periphery.

## Teaser

**Background:** TCR recognition of bipartite ligands composed of self (MHC) and non-self (peptide) maintains T-cell specificity.

**Results:** Mutation of residues in the cognate peptide override TCR mutations that enhance MHC binding.

**Conclusion:** TCR-pMHC binding affinity requires specific TCR-peptide interactions.

**Significance:** Stabilization of TCR-pMHC engagement by TCR-peptide interactions maintains T-cell specificity and prevents recognition of self-pMHC in the periphery.

## Introduction

αβ T-cells protect against pathogens and cellular malignancies by recognizing short peptide fragments bound to major histocompatibility complex (pMHC)^4^ molecules (1, 2). The rigors of T-cell immunity require that TCRs bind to self-pMHC during thymic selection but discriminate between self and non-self-pMHC thereafter by the rapid scanning of huge numbers of potential antigens on the target cell surface (3, 4). Here, we examine how TCR interactions with the variable peptide component of the antigen are balanced against contacts with the MHC to enable T-cells to activate if sensing danger, while remaining tolerant to self.

X-ray crystallographic studies have shown that the αβ TCR docks diagonally across the pMHC class I (pMHCI) peptide binding groove with the TCR α chain contacting the MHCI α2 helix and the TCR β chain contacting the MHCI α1 helix (5). A similar diagonal binding modality has been observed for TCR-pMHC class II interactions with the TCR α chain contacting the MHCII β1 helix and the TCR β chain contacting the MHCI α1 helix. This fixed polarity is conserved in all published TCR-pMHCI structures to date, although the binding angle and contacts between individual TCR-pMHCI complexes can vary substantially (5). TCR recognition of pMHC is mediated through the TCR complementarity-determining region (CDR) loops. Although not the case with all TCR-pMHC pairs (6), the current dogma proposes that the germ line-encoded TCR CDR2 loops contact mainly the conserved helical region of the MHC surface (TCR-MHC self-interaction); the somatically rearranged, hypervariable CDR3 loops contact mainly the antigenic peptide (TCR-peptide non-self interaction), and the CDR1 loops lie in between, contacting both the peptide and the MHC (5).

This binding conformation has led to the suggestion that TCRs contact MHC in a genetically conserved manner (7–10). Indeed, a study by Wu *et al.* (11) investigating the role of the TCR CDR loops during pMHC binding concluded that an initial transition state is formed between the TCR and the MHC surface enabling the TCR to scan the antigenic peptide (two-step binding) (12). This two-step binding model is considered to represent an important mechanism of allowing T-cells to sample a diverse array of pMHC antigens (3, 4, 13). In support of this notion, combined studies have suggested the existence of so-called “interaction codons” that enable the TCR to contact the MHC surface in a conserved manner (7–10). Furthermore, structural comparison of different TCRs with genetically identical CDR2β loops, in complex with the same pMHC, lends support to the idea that some TCRs may use genetically fixed pairwise interactions to bind to the MHC surface (9, 14). In combination, these data predict that interactions between the TCR and MHC stabilize the initial “encounter complex.” However, there is also a body of evidence that contradicts this model of TCR binding (15–21). Thus, there is still much controversy over this central question concerning the nature of T-cell antigen recognition.

The binding affinity of natural TCR-pMHC interactions (*K_D_*∼ 0.1–500 μm) (22, 23) is near the limits of detection using current biophysical techniques. This restricts the scope for investigating TCR-pMHC interactions by mutating important contacts, because altering this weak interaction often results in the loss of any detectable binding using surface plasmon resonance (SPR). To examine the roles of the TCR CDR loops when binding to pMHC, we designed a range of enhanced affinity soluble TCRs with mutations in either their CDR2 and/or CDR3 loops. These unique enhanced affinity reagents enabled investigation of the effects of altering specific interactions between the TCR and MHC or TCR and peptide to examine how individual components of the interface between the TCR and pMHCI contribute to T-cell antigen recognition. These data shed new light on how T-cells might be selected in the thymus to maintain tolerance to self, the mechanism of T-cell cross-reactivity, and the nature of T-cell antigen recognition.

## EXPERIMENTAL PROCEDURES

### 

#### 

##### Generation of Expression Plasmids

A number of constructs were prepared that contained wild type and high affinity TCRs to the HLA A*0201-restricted antigens, Melan-A/MART-1(26–35) ELAGIGILTV (24) and hTERT(540–548) ILAKFLHWL (25). The HLA A*0201-ELAGIGILTV- and HLA A*0201-ILAKFLHWL-specific wild type TCRs (MEL5 and ILA1 TCRs, respectively), the high affinity TCR α and β chains, HLA A*0201 heavy chain, and β2m were generated by PCR mutagenesis (Stratagene) and PCR cloning. All sequences were confirmed by automated DNA sequencing (Lark Technologies). The high affinity HLA A*0201-ELAGIGILTV and HLA A*0201-ILAKFLHWL TCRs were produced using a phage display library as reported previously (26). All of the TCR sequences were constructed implementing a disulfide-linked construct to produce the soluble domains (variable and constant) for both the α (residues 1–207) and β chains (residues 1–247) (27, 28). The HLA A2 heavy chain (residues 1–248) (α1, α2, and α3 domains), tagged with a biotinylation sequence, and β2m (residues 1–100) were also cloned and used to make the pMHCI complexes. The TCR α and β chains, the HLA A2 α chain and β2m sequences were inserted into separate pGMT7 expression plasmids under the control of the T7 promoter (27).

##### Protein Expression, Refolding, and Purification

Competent Rosetta DE3 *Escherichia coli* cells were used to produce the TCR α and β chains, HLA A*0201 heavy chain and β2m in the form of inclusion bodies using 0.5 mm isopropyl 1-thio-β-d-galactopyranoside to induce expression as described previously (27, 29, 30).

##### pMHCI Biotinylation

Biotinylated pMHCI was prepared as described previously (31).

##### SPR Equilibrium Analysis

The binding analysis was performed using a BIAcore T100^TM^ equipped with a CM5 sensor chip as reported previously (32).

##### SPR Kinetic Analysis

Experiments were carried out to determine the *K*_on_ and *K*_off_ values for the TCRs at 25 °C as reported previously (33). Briefly, for all kinetic experiments, ∼300 response units of pMHC were coupled to the CM5 sensor chip surface. The TCR was then injected at concentrations ranging from 10 times above and 10 times below the known *K_D_* value of the interaction at 45 μl/min. The *K*_on_ and *K*_off_ values were calculated assuming 1:1 Langmuir binding (*AB* = *B*·*AB*_max_/(*K_D_* + *B*)), and the data were analyzed using a global fit algorithm (BIAevaluation^TM^ 3.1).

##### SPR Kinetic Titration Analysis

To stringently examine the binding of the TCRs at a greater range of concentrations, we used a new method for analyzing the kinetic parameters of high affinity interactions with long off rates (34). Each TCR was analyzed at five concentrations that represented the greatest range we could accurately achieve around the *K_D_* value of each interaction. During the analysis, ∼300 response units of pMHC were immobilized onto the CM5 sensor chip surface. Each concentration of TCR was injected at a high flow rate of 45 μl/min for a 240-s association period and a 120-s dissociation period. The final and highest concentration had a longer dissociation period of 600 s. A fast flow rate and a low amount of immobilized pMHC were used to limit association and dissociation mass transfer limitations as recommended by the experts at BIAcore^TM^. The *K*_on_ and *K*_off_ values were calculated assuming 1:1 Langmuir binding (*AB* = *B*·*AB*_max_/(*K_D_* + *B*)), and the data were analyzed using the kinetic titration analysis algorithm (BIAevaluation^TM^ Version 3.1) (35).

##### Crystallization and X-ray Data Collection

α1β1-A2-ILA crystals were grown in 20 mm Tris, pH 7.5, 20% PEG 4000, and 10 mm NaCl. All crystals were soaked in 30% ethylene glycol before cryo-cooling. Data were collected at 100 K at the Diamond Light Source, UK. Reflection intensities were estimated with the XIA2 package (36), and the data were scaled, reduced, and analyzed with SCALA and the CCP4 package (37). Structures were solved with molecular replacement using PHASER (38). Sequences were adjusted with COOT (39), and the models were refined with REFMAC5. Graphical representations were prepared with PyMOL (40). The reflection data and final model coordinates were deposited with the PDB database (α1β1-A2-ILA, PDB 4MNQ).

## RESULTS

### 

#### 

##### Design of a Panel of High Affinity TCRs Specific for Two Different HLA A*0201-restricted Peptides

**FIGURE 1.**
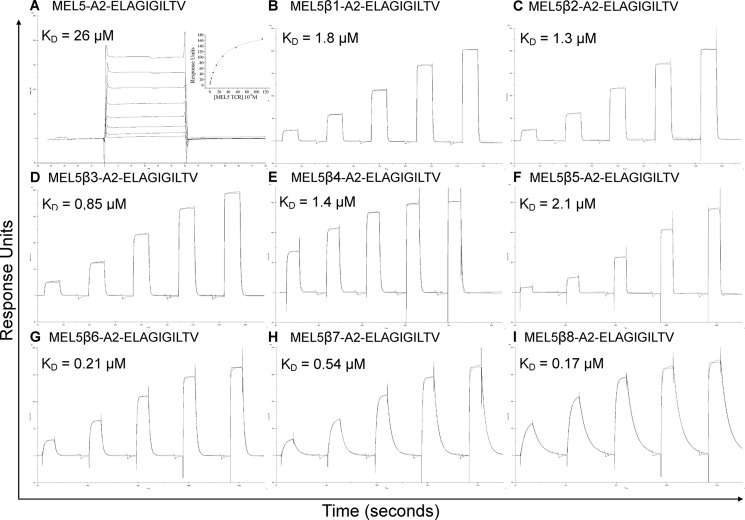
**Affinity and kinetic analysis of wild type and high affinity MEL5-derived TCRs.** *A–I,* these data were produced using a BIAcore T100^TM^ and were then analyzed using equilibrium analysis, kinetic global fit analysis, and kinetic titration analysis. The raw data and the fits are shown in each panel. These data illustrate the improved binding capabilities of the high affinity mutant HLA A2-ELAGIGILTV-specific TCRs compared with the MEL5 TCR. None of the HLA A2-ELAGIGILTV-specific TCRs bound to the HLA A2-ELAGIGILTV with alanine or glycine substitutions.

**FIGURE 2.**
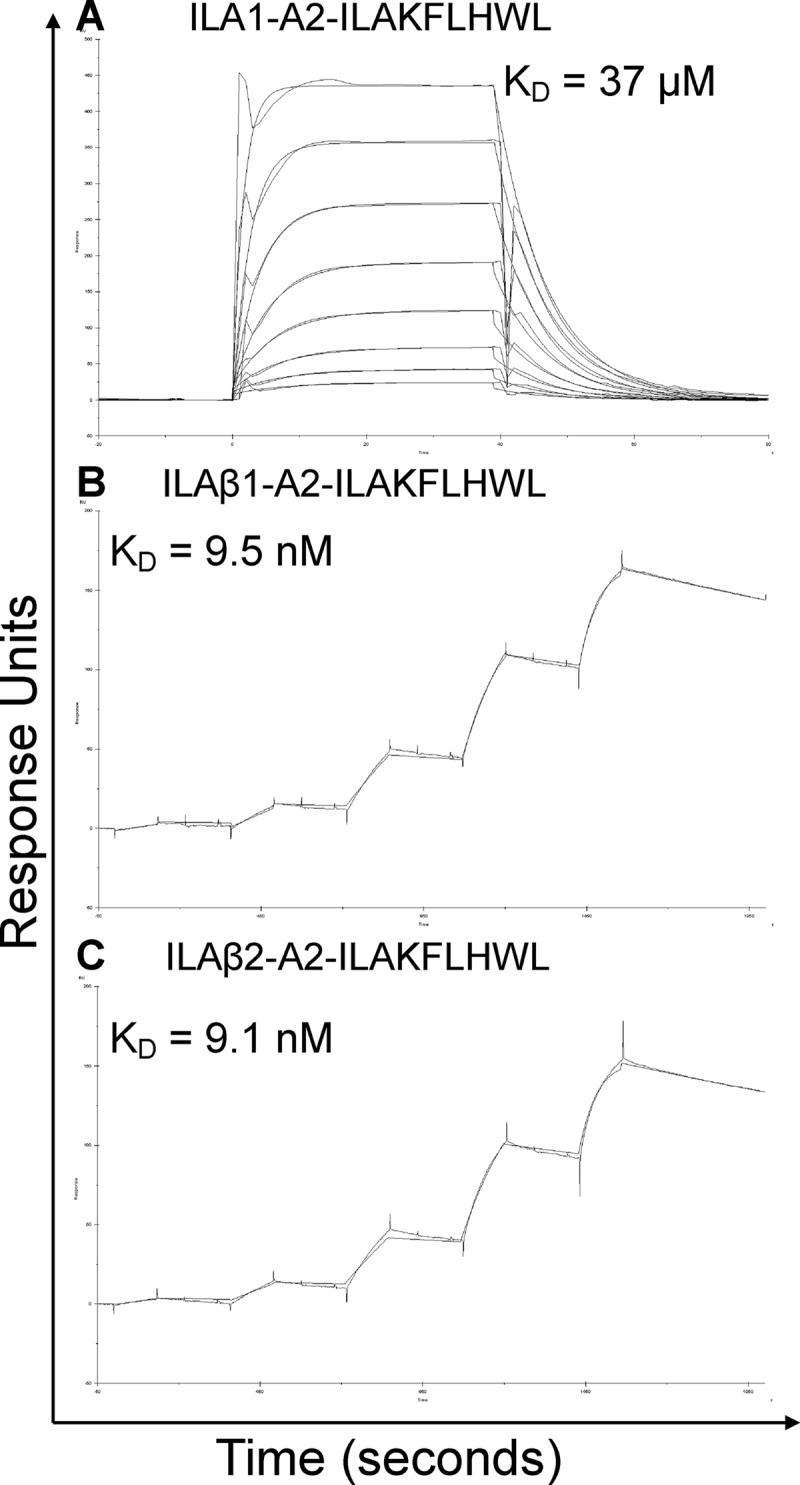
**Affinity and kinetic analysis of wild type and high affinity ILA1-derived TCRs.** *A–C,* these data were produced using a BIAcore T100^TM^ and were then analyzed using equilibrium analysis, kinetic global fit analysis, and kinetic titration analysis. The raw data and the fits are shown in each panel. These data show that the ILA1β1 and ILA1β2 TCRs bound to HLA A*0201-ILAKFLHWL with ∼4000 times greater affinity than the wild type ILA1 TCR.

**FIGURE 3.**
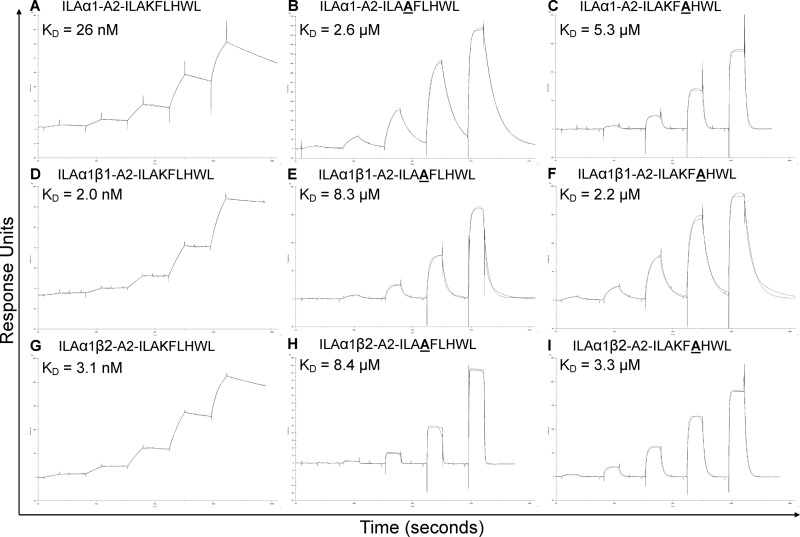
**Effect of alanine peptide substitutions on high affinity ILA1-derived TCR binding.** Binding affinity and kinetic analysis of the HLA A*0201-ILAKFLHWL-specific high affinity ILA1α1, ILA1α1β1, and ILA1α1β2 TCRs to HLA A2-ILAKFLHWL, HLA A2-ILA**A**FLHWL, and HLA A2-ILAKF**A**HWL (*A–I*). These data were produced using a BIAcore T100^TM^ and were then analyzed using equilibrium analysis, kinetic global fit analysis, and kinetic titration analysis. The raw data and the fits are shown in each panel. These data show the effect of the HLA A2-ILA**A**FLHWL and HLA A2-ILAK**A**LHWL peptide modifications on the binding of the high affinity TCRs compared with HLA A2-ILAKFLHWL. These support the notion that TCR-peptide interactions govern TCR-pMHC binding because, although the ILA1α1β1 TCR with a mutated CDR2 loop did not contact the peptide, the difference in binding between the ILA1α1 TCR and the ILA1α1β1 TCR to HLA A2-ILA**A**FLHWL and HLA A2-ILAK**A**LHWL compared with HLA A2-ILAKFLHWL is disproportionately different.

Investigating the individual roles of the TCR CDR loops when binding to pMHC has been difficult because the weak binding affinities of natural TCR-pMHC interactions (0.1–500 μm) (22, 23) are close to the limits of detection by SPR. To overcome this problem, we designed a range of soluble TCRs with up to 18,500-fold enhancement in affinity for cognate antigen using CDR loop mutations selected by phage display (Table 1) (26). We first measured the binding affinity and kinetics of wild type and enhanced affinity TCRs specific for either the HLA A*0201-restricted peptide antigens, Melan-A/MART-1(26–35) (ELAGIGILTV), or hTERT(540–548) (ILAKFLHWL). The HLA A*0201-ELAGIGILTV-specific TCRs with mutated CDR2 or CDR2 and CDR3 loops bound with considerably stronger affinities (*K_D_*s between 12 and 897 times greater) than the parent wild type TCR (MEL5) (Fig. 1 and Table 2). The HLA A*0201-ILAKFLHWL specific TCRs with mutated CDR2 loops bound to cognate antigen with a substantially stronger affinity (*K_D_* values between 1423 and 4066 times greater) compared with the parent wild type TCR (ILA1) (Fig. 2 and Table 3). Similarly, when the mutated CDR2 and CDR3 loop mutations were combined, we observed a further increase in binding affinity (*K_D_* values up to 18,500 times greater than ILA1 TCR), indicating that the mutations could be used cooperatively (Fig. 3 and Table 3). In agreement with our previous findings (26, 34, 41–44), enhanced TCR affinity was due to small increases in the on-rate, and vastly extended off-rates (Tables 2 and 3, Figs. 1–3). The stronger affinities of the high affinity TCRs enabled the modulation of individual components of the TCR-peptide interaction through peptide mutations while maintaining enough residual binding to detect using SPR in later experiments.

**TABLE 1:**
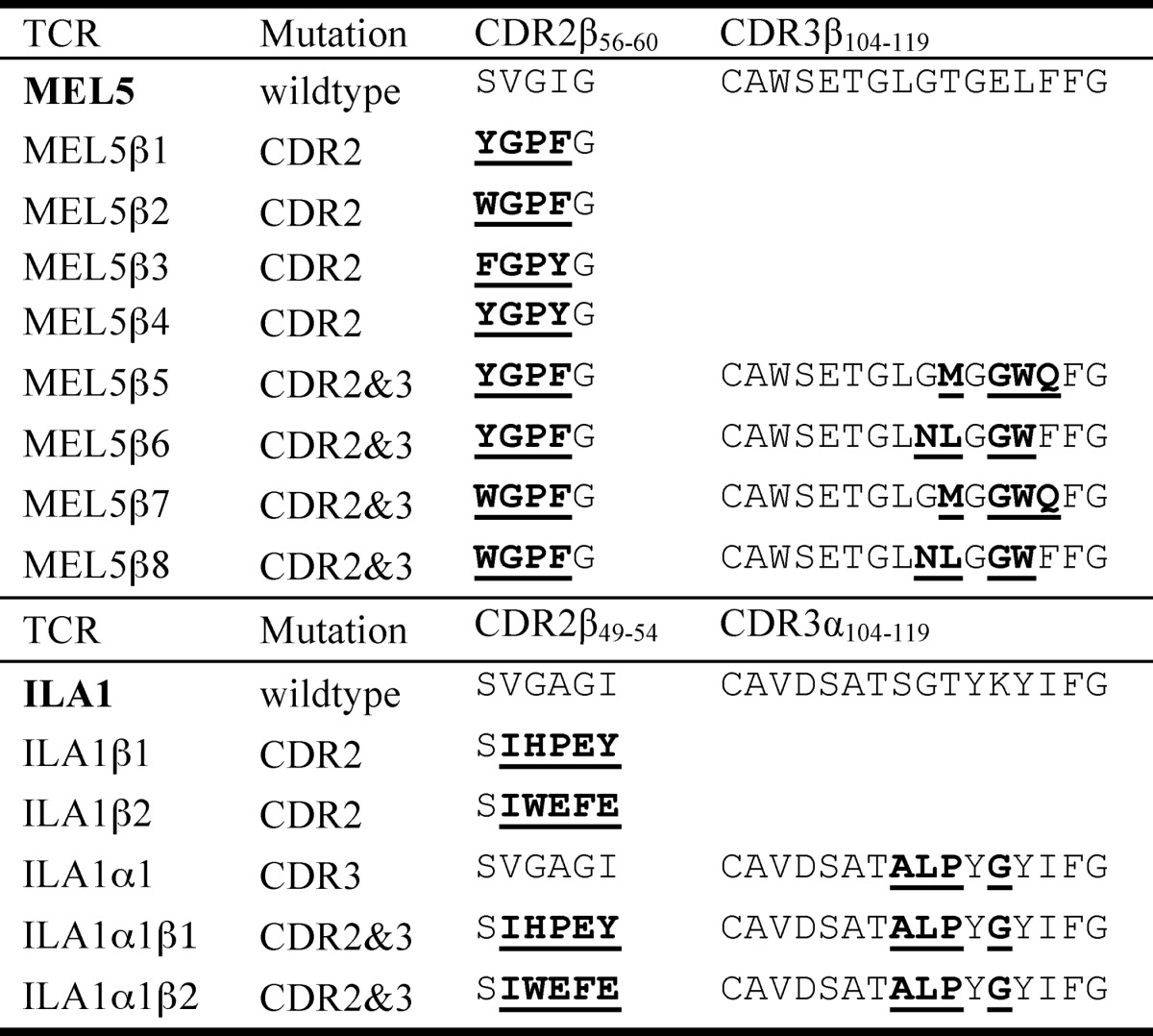
**Sequence comparison of high affinity TCRs**

**TABLE 2:**
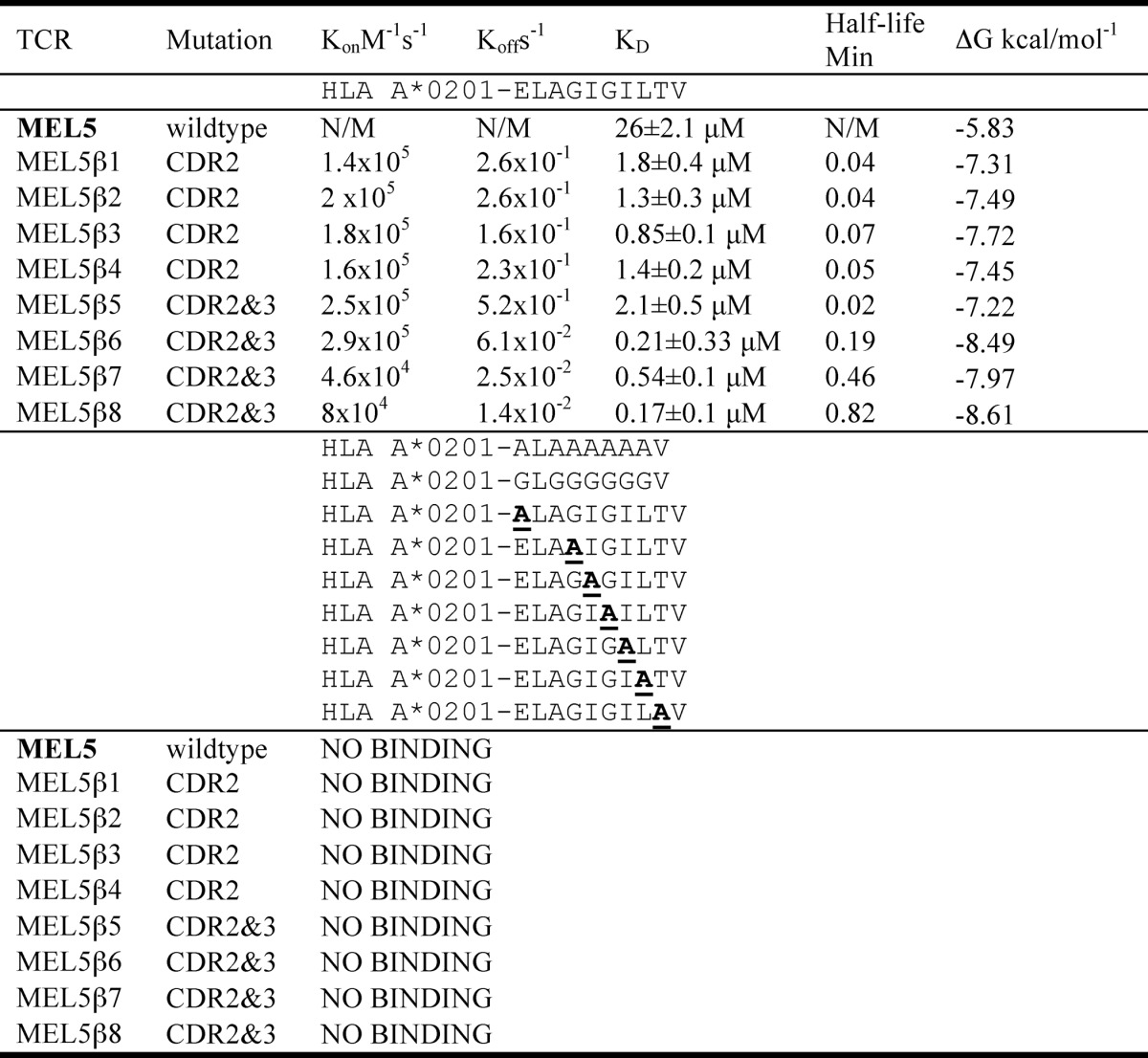
Kinetic and affinity analysis of high affinity HLA-A*0201-ELAGIGILTV-specific TCR binding to alanine- and glycine-substituted peptides

**TABLE 3:**
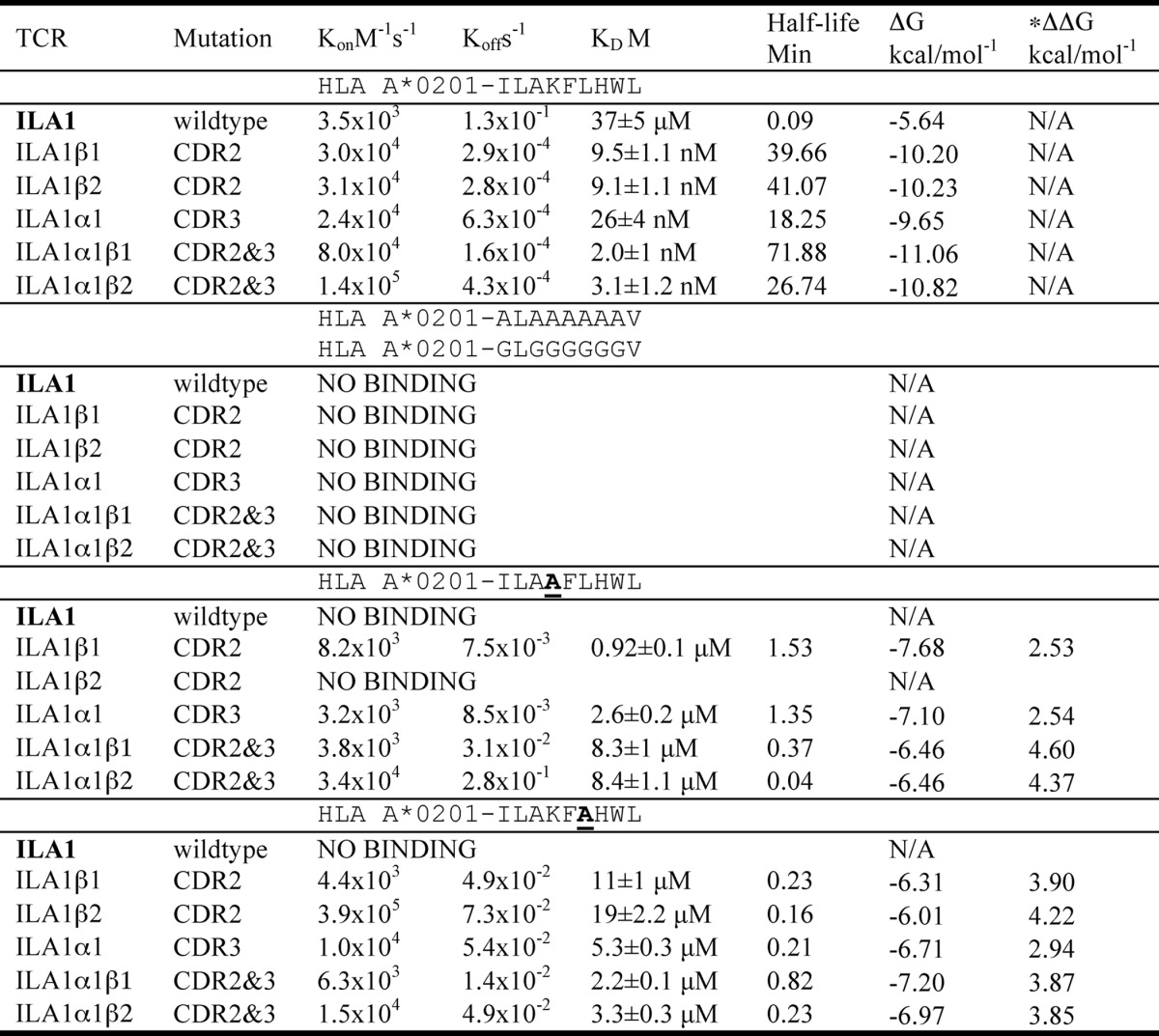
Kinetic and affinity analysis of high affinity HLA-A*0201-ILAKFLHWL-specific TCRs binding to alanine- and glycine-substituted peptides

* ΔΔ*G*^0^ = Δ*G*^0^ of each TCR binding to HLA A2-ILAKFLHWL alanine variants minus Δ*G*^0^ of TCR to HLA A2-ILAKFLHWL.

##### High Affinity CDR2 Loop Mutated TCRs Do Not Bind to “Null” Peptides

**FIGURE 4.**
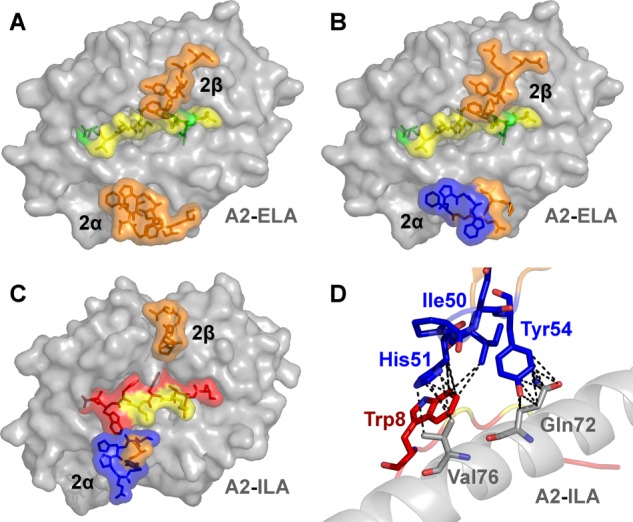
**Peptide modifications do not directly impinge on the binding of mutated high affinity TCR residues.** The complex structures of high affinity MEL5- and ILA1-derived TCRs show that peptide modifications do not directly impinge on the binding of mutated high affinity TCR residues. *A,* wild type MEL5-A2-ELA (PDB code 3HG1 (24)) complex structure showing the MHC in *gray surface*, the mutated peptide residues in *yellow stick* and *surface* (nonmutated residues in *green*), and the positions of the TCR CDR2 loops in *orange sticks* and *surface. B,* high affinity α24β17-A2-ELA (PDB code 4JFF (34)) complex structure (α24β17 is a high affinity version of the MEL5 TCR) showing the MHC in *gray surface*, the mutated peptide residues in *yellow stick* and *surface* (nonmutated residues in *green*), and the positions of the TCR CDR2 loops in *orange sticks* and *surface*. In this structure, the high affinity mutations in the TCR CDR2α loop are colored *blue* and are distal from the peptide. *C,* high affinity α1β1-A2-ILA (PDB code 4MNQ) complex structure (α1β1 is a high affinity version of the ILA1 TCR) showing the MHC in *gray surface*, the mutated peptide residues in *yellow stick* and *surface* (nonmutated residues in *red*), and the positions of the TCR CDR2 loops in *orange sticks* and *surface. D,* specific contacts between the high affinity mutated residues in the α1β1 TCR CDR2α loop (*blue sticks*), the MHC (*gray sticks*), and the peptide (*red*). The peptide residues that were mutated to alanine (*yellow*) were not directly contacted by the high affinity mutated residues in the α1β1 TCR CDR2α loop. Overall, these structures demonstrate that TCR residues that have been mutated in the CDR2 loops of high affinity TCRs do not directly contact residues that were mutated to alanine in the peptide.

Our recent structure of a high affinity variant of the MEL5 TCR (34) demonstrated that the mutated CDR2α region of the high affinity MEL5-derived TCRs used in this study was in an identical position to the wild type MEL5 TCR (24), distal from the peptide (Fig. 4, *A* and *B*). Thus, we concluded that mutations at residues in the peptide were very unlikely to directly affect the high affinity interactions between the MEL5 derived high affinity TCRs and the MHC surface. We reasoned that, because the general dogma of TCR engagement postulates that TCR-MHC interactions bind before TCR-peptide sampling, the high affinity interaction between the MEL5-derived TCRs and the MHC surface should retain some measurable ability to bind to the surface of HLA A2 irrespectively of the bound peptide, because the interaction between the TCR and the peptide should only account for a small proportion of the overall binding energy (δG). To investigate the role of peptide modifications on TCR-pMHC docking, we manufactured two null HLA A*0201-nonamer peptide complexes where nonprimary MHC anchors were substituted with either glycine or alanine. However, we were unable to detect binding of any of the HLA A*0201-ELAGIGILTV- or HLA A*0201-ILAKFLHWL-specific high affinity TCRs tested against the HLA A*0201-GLGGGGGGV or HLA A*0201-ALAAAAAAV null antigens (Tables 2 and 3). These observations are remarkable when considering that, for example, the CDR2 loop modified ILA1β2 TCR bound to HLA A*0201-ILAKFLHWL with an affinity >4000 times greater than the wild type ILA1 TCR (Table 3 and Fig. 2). These data support the notion that specific interactions between the TCR and peptide are required to allow the TCR to effectively engage MHC and demonstrate that changes to the antigenic peptide override mutations in the TCR that enhance contacts with the MHC molecule.

##### CDR2-mutated High Affinity HLA A*0201-ELAGIGILTV-specific TCRs Are Extraordinarily Sensitive to Peptide Substitutions

To investigate the role of more conservative peptide modifications on TCR binding affinity, we introduced single alanine substitutions into the HLA A*0201-ELAGIGILTV (MART-1/Melan A-derived) peptide antigen. We substituted residues in the peptide that were very unlikely to directly affect the high affinity regions of the MEL5-derived high affinity TCRs and the MHC surface, according to our structural evidence (Fig. 4, *A* and *B*) (24, 34). This enabled the determination of the effect of altering TCR-peptide contacts on TCR-MHC binding. Neither the MEL5 TCR nor any of the high affinity TCRs retained the ability to bind to HLA A*0201-ELA**A**IGILTV, HLA A*0201-ELAGI**A**ILTV, HLA A*0201-ELAGIG**A**LTV, or HLA A*0201-ELAGIGIL**A**V (Table 2). This observation that single alanine substitutions in the native peptide can completely abrogate binding of all of the high affinity HLA A*0201-ELAGIGILTV-specific TCRs reaffirms the notion that specific interactions between the TCR and peptide are required in order for optimal docking with the MHC surface to occur, and this is consistent with our previous findings using this system (34).

##### Affinity-enhanced CDR2α Loops of the ILA1-derived TCRs Do Not Contact Alanine-substituted Peptide Residues

**FIGURE 5.**
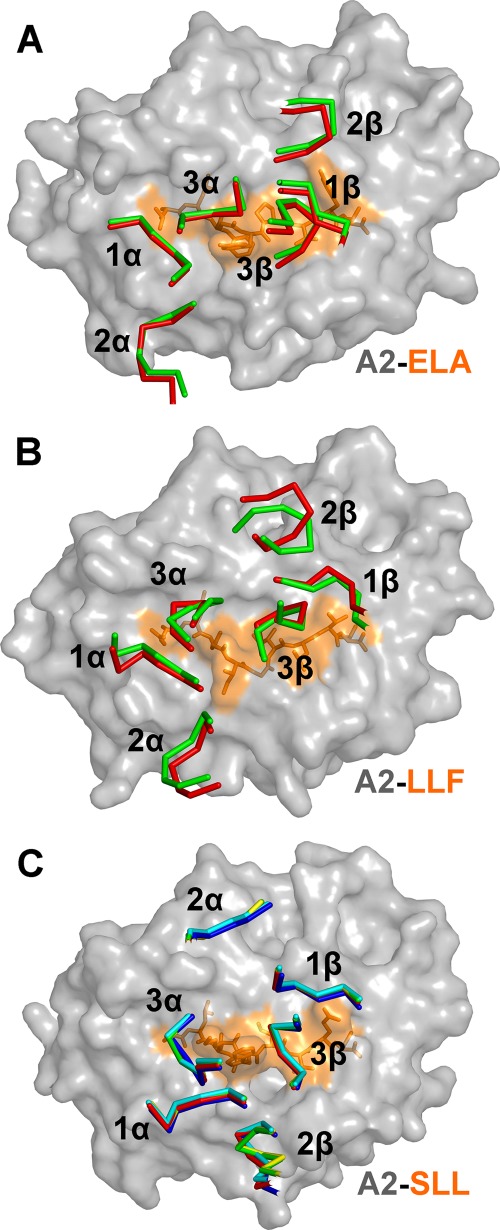
**Conformation of TCR CDR loops remains very similar between modified high affinity TCRs and their wild type progenitors.** Comparison of the CDR loop positions of previously published high affinity and wild type TCRs. *A,* wild type MEL5-A2-ELA complex (24) (CDR loops in *orange ribbon*) and the high affinity α24β17-A2-ELA complex (34) (CDR loops in *green ribbon*). *B,* wild type A6-A2-LLF complex (27) (CDR loops in *orange ribbon*) and the high affinity c134-A2-LLF complex (41) (CDR loops in *green ribbon*). *C,* wild type 1G4-A2-SLL complex (64) (CDR loops in *orange ribbon*) and the high affinity c58c62-A2-SLL (CDR loops in *green ribbon*), c49c50-A2-SLL (CDR loops in *blue ribbon*), c549c61-A2-SLL (CDR loops in *yellow ribbon*), and c5c1-A2-SLL (CDR loops in *cyan ribbon*) complexes (42, 43). In all cases, the relative positions of the CDR loops over the pMHC for the wild type TCRs and their high affinity TCR derivative are virtually identical.

Previous structural comparisons of wild type and high affinity TCRs (34, 41–43) show that these molecules adopt a near identical binding mode (Fig. 5). To confirm that alanine substitutions of these peptide residues would not directly impinge on the mutated residues in the high affinity CDR2α loops of the ILA1-derived TCRs, we solved the structure of the ILA1α1β1 TCR in complex with HLA A*0201-ILAKFLHWL. The complex was solved to a resolution of 2.4 Å in space group C121. The resolution was sufficiently high to show that the interface between the two molecules was well ordered and contained well defined electron density. The crystallographic *R*/*R*_free_ factors were 20.1 and 24.6%, within the accepted limits shown in the theoretically expected distribution (Table 4) (45). The structure demonstrated that the mutated CDR2α region of the high affinity α1β1 TCR could not directly contact the mutated residues in the peptide (Fig. 4, *C* and *D*, and Table 5).

**TABLE 4:** **Data collection and refinement statistics for α1β1-A2-ILA complex structure** One crystal was used for solving the structure. Values in parentheses are for the highest resolution shell.

	α1β1-A2-ILA
PDB code	4MNQ
**Data collection**	
Space group	P3_2_21
Cell dimensions	
*a*, *b*, *c*	97.14, 97.14, 123.08 Å
α, β, γ	90, 90, 120°
Resolution (Å)	49.7 to 2.4 Å (10.7 to 2.4 Å)
*R*_merge_	19.2%
*I*/σ*I*	16.6
Completeness	100%
Redundancy	10.9

**Refinement**	
Resolution	2.4 Å
No. of reflections	25,403
*R*_work_/*R*_free_	20.1/24.6
No. of atoms	3694
Protein	3492
Ligand/ion	41
Water	161
*B*-factors	44.63
Protein	44.60
Ligand/ion	60.79
Water	41.10
Root mean square deviations	
Bond lengths	0.022 Å
Bond angles	1.206°

**TABLE 5:**
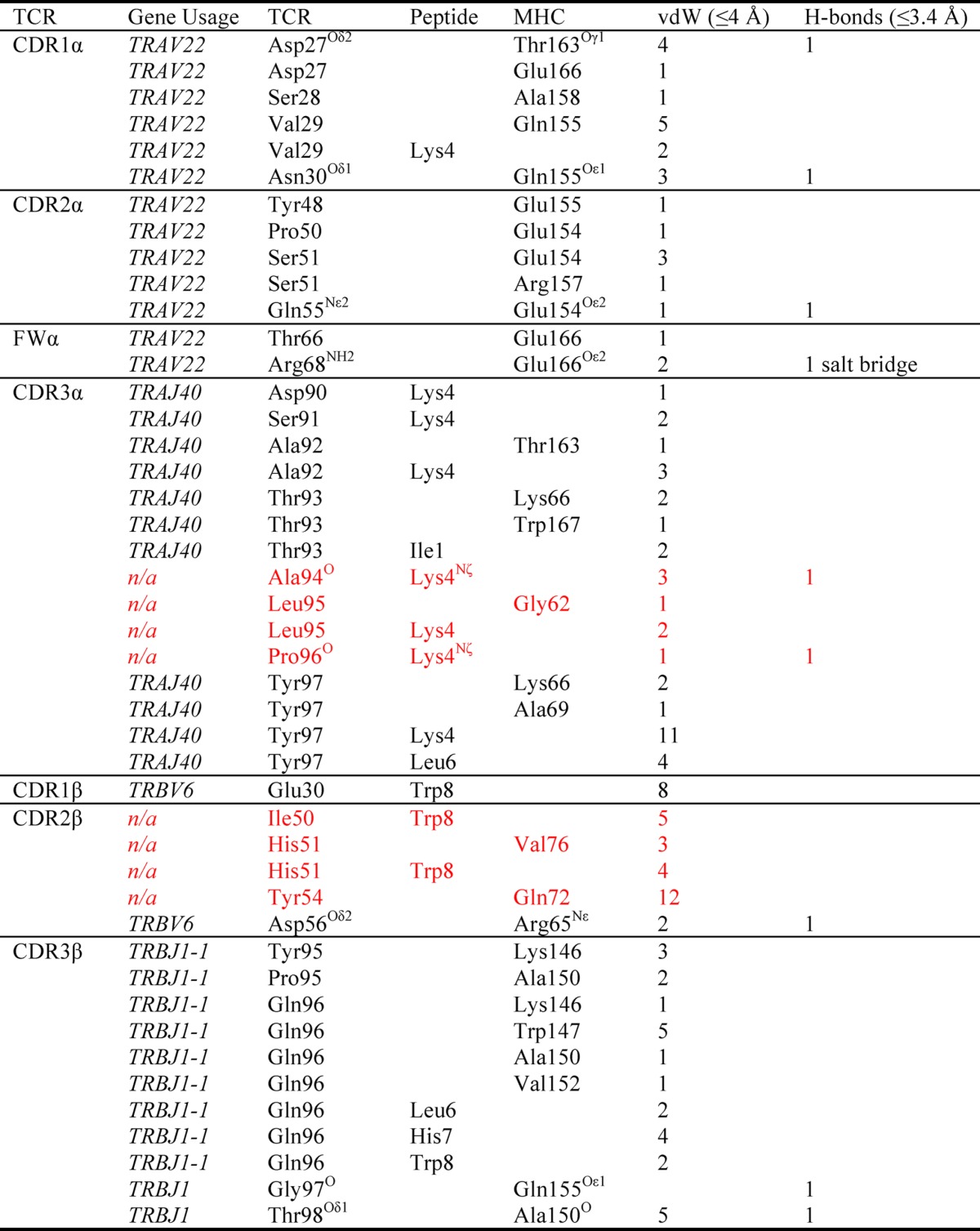
**α1β1-A2-ILA contacts (residues mutated from wild type shown in red)**

* A 3.4-Å cutoff was used for H-bonds and salt bridges, and a 4-Å cutoff was used for van der Waals (vdW).

##### HLA A*0201-ILAKFLHWL Peptide Substitutions Disproportionately Affect the Binding of High Affinity CDR2 Loop Mutated TCRs

**FIGURE 6.**
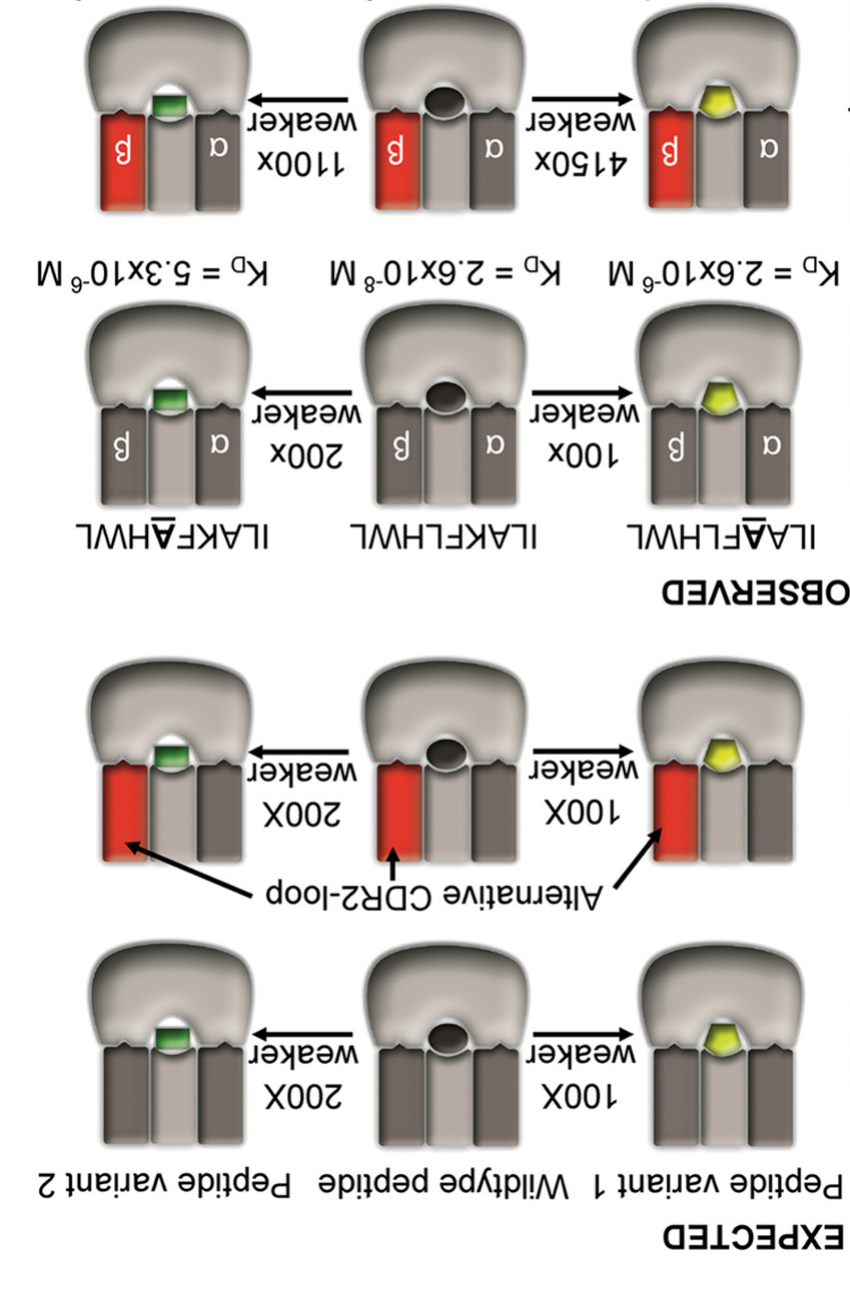
**Schematic of the effect of alanine peptide substitutions on TCR-pMHC binding affinity.** Nonmutated TCR and pMHC components are shown in grayscale. TCRs with high affinity mutations are shown in *red*. Peptide mutations are shown in *yellow* or *green. A,* expected difference in the binding of an unmodified TCR (TCR1) compared with a CDR2 loop mutated TCR (TCR2) (mutation shown in *red*), assuming that the CDR2 loops bind independently of the TCR-peptide interaction. Because the mutated CDR2 loop (shown in *red*) does not contact the peptide, the theoretical difference in binding between the TCR1 and TCR2 to peptide variants 1 (*yellow*) and 2 (*green*) compared with the wild-type peptide (*black*) should be identical according the interaction between the CDR3 loops and the peptide. *B,* schematic of the observed difference in the binding of the ILA1 α1β1 TCR, compared with the ILA1 α1 TCR engaging a peptide-MHC complex. These data show that a disproportionate knock-on effect in binding occurs for the ILA1 α1β1 TCR, compared with the ILA1 α1 TCR. These data indicate that TCR-MHC binding does not occur independently of TCR-peptide interactions and that the latter likely governs the former.

We then measured the binding of the HLA A*0201-ILAKFLHWL-specific wild type ILA1 TCR and high affinity derivative TCRs to HLA A*0201-ILA**A**FLHWL and HLA A*0201-ILAKF**A**HWL (Table 3 and Fig. 3). The ILA1α1 high affinity TCR bound to HLA A*0201-ILA**A**FLHWL and HLA A*0201-ILAKF**A**HWL with 100 and 200 times weaker affinity, respectively, than to HLA A*0201-ILAKFLHWL. This difference corresponded to a ΔΔ*G* value (difference in binding energy, Δ*G*, between the ILA1α1 high affinity TCR interacting with HLA A*0201-ILAKFLHWL *versus* HLA A*0201-ILA**A**FLHWL and HLA A*0201-ILAKF***A***HWL) of 2.54 and 2.94 kcal/mol^−1^, respectively. We then repeated this analysis using the ILA1α1β1 and ILA1α1β2 TCRs. These TCRs contained modified CDR2 loops but identical CDR3 loops to the ILA1α1 TCR. According to the assumption that TCR-MHC interactions precede TCR-peptide interactions, we reasoned that each of these TCRs should retain their individual TCR-MHC contacts because only the TCR-peptide interaction should be directly affected (as in Fig. 6*A*). Therefore, the difference in binding affinity observed for the ILA1α1 TCR between HLA A*0201-ILAKFLHWL compared with HLA A*0201-ILA**A**FLHWL and HLA A*0201-ILAKF**A**HWL (100 and 200 times weaker affinity, respectively) should be similar to the ILA1α1β1 and ILA1α1β2 TCRs. However, the ILA1α1β1 TCR bound to the HLA A*0201-ILA**A**FLHWL and HLA A*0201-ILAKF**A**HWL with 4150 and 1100 times weaker affinity (ΔΔ*G* value of 4.6 and 3.87 kcal/mol^−1^), respectively (Table 3 and Figs. 3 and 6*B*). The ILA1α1β2 TCR bound to HLA A*0201-ILA**A**FLHWL and HLA A*0201-ILAKF**A**HWL with 2710 and 1065 times weaker affinity (ΔΔ*G* value of 4.37 and 3.85 kcal/mol^−1^), respectively (Table 3 and Fig. 3). These data demonstrate that TCR interactions with the peptide and MHC are strongly coupled and that modifying the TCR-peptide interaction has a disproportionately strong detrimental energetic effect on TCR-MHC binding.

##### Effect of Peptide Substitutions on the Binding Kinetics of High Affinity CDR2 Loop Mutated TCRs

Kinetic binding analyses were carried out at 25 °C to measure the on-rate (*K*_on_) and off-rate (*K*_off_) for each TCR-pMHCI interaction (Figs. 1–3 and Tables 2 and 3). These analyses were important to reveal the kinetic basis for the effect of altering the TCR-peptide interactions by modifying the antigenic peptide. Interestingly, although alanine mutations within the central peptide residues reduced the binding affinity of all of the high affinity TCRs tested to a different extent (97–4150-fold reduction in binding affinity), the on-rate was not substantially affected (average decrease of 10 times) (Tables 2 and 3). Conversely, the stability of the TCR-pMHC complex was affected by a greater extent, as evident by the faster off-rate observed (average increase of 200 times) (Tables 2 and 3). Therefore, a faster off-rate was the major kinetic determinant governing the decrease in binding affinity between the high affinity CDR loop mutated TCRs and the alanine-substituted peptide ligands. These data suggest that in order for the TCR to form a stable long lived interaction with cognate pMHC, the TCR must be able to bind to the peptide to allow optimal MHC docking. Thus, in the systems we have studied, successful TCR-peptide sampling must precede (or occur at the same time as) the stabilizing interaction between the TCR and the MHC surface.

## DISCUSSION

Antigen recognition by the TCR usually involves contacts with both self (MHC) and non-self (the antigenic peptide) (5, 16). To avoid autoreactivity, the self-interaction between the TCR and MHC must not be sufficient to activate peripheral T-cells independently of the non-self TCR-peptide interaction. The current database of TCR-pMHC complex structures shows that the interaction between TCR and antigenic peptide can play a minimal structural role, often being responsible for less than a third of the binding interface relative to contacts between the TCR and MHC (5). Thus, the molecular mechanism by which the TCR maintains peptide-specific recognition is not immediately obvious.

To re-examine how the TCR CDR loops co-operatively act to stabilize TCR-pMHC binding, we designed a range of soluble TCRs that exhibited up to a 18,500-fold enhancement in affinity for cognate antigen using CDR loop mutations selected by phage display (26). Previous studies using high affinity TCRs have shown that these artificial reagents retain a high level of antigen specificity similar to their wild type progenitors (34, 46–48). In all cases, the enhanced affinity observed for the mutated TCRs compared with the wild type TCRs was due to small differences in the on-rate but a vastly extended off-rate. The slower off-rate indicated that any initial “transition state” was less important than the formation of a stable complex during high affinity TCR-pMHC binding. These high affinity TCRs enabled modification to the TCR-peptide interaction while retaining a strong enough residual TCR-pMHC affinity to measure using SPR. Furthermore, we were able to incorporate mutations into individual CDR loops to generate a panel of TCRs with an identical sequence except for their CDR2 loops. Structural analyses confirmed that these loops were distal to peptide binding in both wild type and enhanced affinity MEL5 and ILA1 TCRs. Based on some models of TCR engagement (11), we reasoned that HLA A*0201-restricted TCRs with high affinity mutations in their CDR2 loops should retain a residual ability to bind to the surface of HLA A*0201 independently of the peptide because the TCR-peptide interactions should only account for a small proportion of the overall binding energy. In contrast to this prediction, we were unable to show binding to the HLA A*0201-GLGGGGGGV or HLA A*0201-ALAAAAAAV null antigens with any of the CDR2 loop high affinity TCRs tested. These observations support the notion that specific interactions between the TCR and peptide are required to allow the TCR to effectively engage MHC. Using this system, we were also able to examine whether subtle alterations in the interaction between TCR and peptide were independent of TCR CDR2 loop binding to MHC. To investigate this, we tested the binding affinity of a panel of HLA A*0201-ILAKFLHWL-specific CDR2 loop-modified TCRs to peptides that contained alanine substitutions at positions structurally shown to be key TCR contacts. These data revealed that even minimal changes to the TCR-peptide interaction had a substantial impact on the TCR affinity and binding energy (Δ*G*). These data show that TCR-peptide contacts are strongly “coupled” to TCR-MHC contacts.

We also performed a kinetic investigation of the effect of altering the TCR-peptide interaction. These data showed that the vastly extended off-rates that governed the enhanced affinity of the high affinity mutated TCRs were effectively nullified by altering the TCR interaction with peptide, although the on-rates remained relatively unchanged. Thus, our data indicate that complex formation is not initiated by TCR-MHC binding. Rather, successful TCR-peptide sampling must precede or occur at the same time as the stabilizing interaction between the TCR and the MHC surface. In support of this notion, our data show that altering the TCR interaction with peptide can override the optimal formation of TCR-MHC interactions resulting in a disproportionate knock-on effect on TCR-pMHC affinity.

**FIGURE 7.**
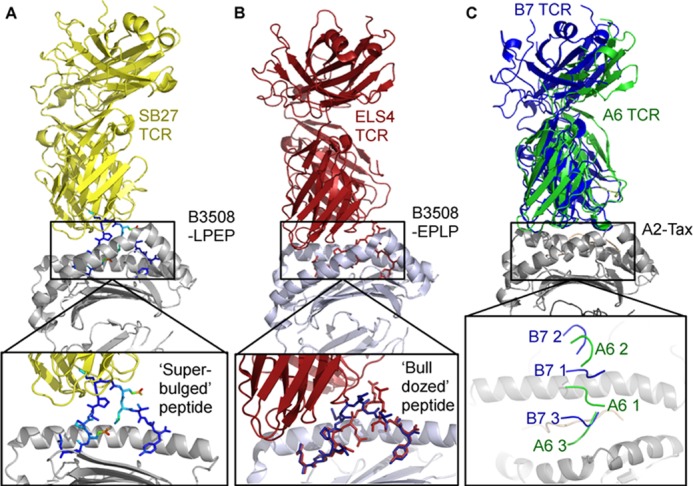
**Structural evidence demonstrating that TCR-peptide contacts precede TCR-MHC contacts.** *A,* co-crystal structure of the SB27 TCR (shown as *yellow schematic*) bound to the HLA-B*3508 (shown as *gray yellow schematic*) LPEP super-bulged 13-mer peptide (shown as *sticks*, colored using Wilson “B” factor) complex. The *expanded panel below* illustrates the extended conformation of the peptide, making it highly improbable that the SB27 TCR could contact the MHC surface before the peptide (19). *B,* co-crystal structure of the ELS4 TCR (shown as *red yellow schematic*) bound to the HLA-B*3501 (shown as *gray yellow schematic*) EPLP 11-mer peptide (shown as *sticks*, colored using *red* complexed to the ELS4 TCR and in *blue* uncomplexed). The *expanded panel below* illustrates how the EPLP peptide is bulldozed into a different conformation during TCR binding (before TCR binding is shown in *blue* and after TCR binding is shown in *red*), allowing the TCR to contact the MHC surface (20). *C,* co-crystal structure of the A6 TCR (shown as *green schematic*) superposed with the B7 TCR (shown as *blue schematic*) which both bind to the HLA A*0201 (shown as *gray schematic*) Tax (shown as *peach schematic*) complex (27, 51, 52). These TCRs share the same β-chain germ line-encoded CDR1 and CDR2 loops, and they bind to the same N-terminal region of the A2-Tax complex. The *expanded panel below* illustrates that the CDR3 loops engage some of the same residues of the peptide, whereas the CDR1 and CDR2 loops bind to distinct regions of the MHC surface.

Mounting evidence from other studies also contests the notion that conserved interactions between the germ line-encoded loops of the TCR and the MHC initiate TCR-pMHC complex formation. First, Burrows *et al.* (16) have demonstrated that disrupting conserved interactions between the TCR and MHC surface resulted in the formation of compensatory interactions. In support of these data, Dyson and co-workers (49) extensively diversified CDR1 and CDR2 loops *in vivo* and demonstrated that the TCR is not genetically hardwired to engage MHC ligands. Second, a number of molecular studies are incompatible with TCR-MHC initiated binding. These include the following: (i) the co-crystal structure of a TCR bound to MHCI complexed with a 13-mer “super-bulged” peptide (19) showing that the extended central peptide bulge physically restricted the TCR from contacting the MHC surface (Fig. 7*A*) (19); (ii) the co-crystal structure of a TCR bound to MHCI complexed with an 11-mer peptide demonstrating that the peptide was “bulldozed” or flattened by the TCR, allowing the TCR to contact the MHC surface (Fig. 7*B*) (20); (iii) accumulated studies showing that TCR-MHC interactions can play a minimal energetic role, compared with TCR-peptide interactions, during TCR binding to MHC (15, 18, 21, 50), and (iv) the structures of the A6 and B7 TCRs bound to HLA A*0201-LLFGYPVYV (27, 51, 52), showing that, despite both TCRs sharing a genetically identical germ line-encoded Vβ-gene (Vβ6-5), the TCR-MHC contacts were distinct, although a number of identical TCR-peptide contacts existed (Fig. 7*C*). Finally, our data, in which we have tested affinity-enhanced TCRs against a range of normal tissue cell samples, show that high affinity CDR2 mutations do not render the TCRs more unspecific than high affinity CDR3 mutations. The examples above are consistent with a model for T-cell antigen recognition in which TCR-peptide binding overrides TCR-MHC engagement.

The idea that TCR-peptide contacts govern T-cell antigen recognition is in accord with several biological requirements of T-cell immunity. First, given that extremely weak TCR binding is required for positive selection of peptide-dependent T-cells in the thymus (53), control of this delicate aspect would represent a far greater challenge were TCR-MHC contacts to proceed TCR-peptide interactions (11). Second, accumulated studies that have demonstrated that alloreactive TCR recognition is peptide-dependent (54–56) are favored by models where TCR-peptide contacts dominate TCR engagement. Third, if TCR-MHC interactions initiate antigen recognition then the extraordinarily rapid kinetics of CD8 and CD4 coreceptor binding might enable aberrant T-cell signaling, bypassing antigen-specific TCR-peptide sampling (57). Fourth, a system where TCR-MHC contacts dominate TCR binding is difficult to reconcile with the kinetic segregation model of T-cell activation (58, 59). In this model, small molecules such as CD2 and CD28 facilitate contact zones to enable the TCR to scan pMHCs. The proximity of the T-cell and target cell membranes in these contact zones excludes large phosphatase molecules, such as CD45, triggering phosphorylation of the TCR and downstream signaling events. Thus, TCR-MHC binding in these contact zones could enable TCR phosphorylation independently of TCR-peptide binding. Finally, a mode of action that requires that the TCR interacts with MHC prior to peptide scanning wastes both time and energy. This is particularly important for a system that requires an individual TCR to scan a multitude of pMHC molecules to locate a cognate peptide.

**FIGURE 8.**
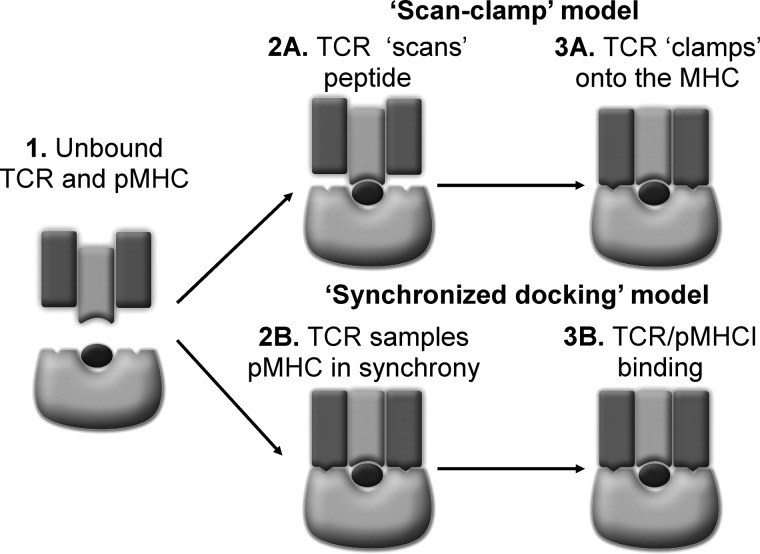
**New models for TCR engagement of pMHC.** *1*. schematic of a TCR (*dark* and *light gray*) proceeding engagement of peptide (*black*)-MHC (*light gray*). *A,* “Scan-clamp” model. Only specific TCR-peptide contacts (*light gray* and *black*) (*2A*) allow the TCR (shown in *dark gray*) to clamp-onto the MHC surface and (*3A*) complete TCR-pMHC docking, which leads to T-cell activation. *B,* “synchronized docking” model. TCR contacts the peptide and MHC simultaneously (*2B*), but TCR-peptide interactions are dominant over TCR-MHC interactions (*3B*). Only the scan clamp and synchronized docking models for T-cell antigen recognition are permissive with our data.

We propose two new models of TCR-pMHC binding that are accommodated by our data and are both temporally and energetically complementary with a system requiring recognition of self in the thymus and rapid intolerance of non-self in the periphery. First, the “scan-clamp” model, in which the TCR “scans” the peptide before “clamping” onto the MHC surface (Fig. 8*A*). Second, the “synchronized docking” model, in which there is no temporal separation between the TCR binding to the peptide or MHC, but TCR-peptide interactions are dominant over TCR-MHC interactions (Fig. 8*B*). These new models are consistent with the requirement for T-cells to target cells based on their antigenic peptide, allowing them to expeditiously distinguish aberrant cells from healthy cells (60–63).

In conclusion, it is clear that T-cells have evolved to ensure that TCR-pMHC binding is carefully balanced to guarantee that the fidelity of antigen recognition is permissive for the conserved and universal interactions that lead to T-cell activation. Our new data shed light on the mechanisms controlling the seemingly paradoxical observation that a receptor-ligand (TCR-pMHC) interaction with both a self (TCR-MHC) and non-self (TCR-peptide) component can control T-cells by only forming productive interactions when encountering alien antigen.

## Footnotes

4 The abbreviations used are:

pMHC: peptide-major histocompatibility complex
SPR: surface plasmon resonance
TCR: T-cell receptor
CDR loop: complementarity-determining region loop
PDB: Protein Data Bank
MHCI: pMHC class I.
